# The International Study of Childhood Obesity, Lifestyle and the Environment (ISCOLE): design and methods

**DOI:** 10.1186/1471-2458-13-900

**Published:** 2013-09-30

**Authors:** Peter T Katzmarzyk, Tiago V Barreira, Stephanie T Broyles, Catherine M Champagne, Jean-Philippe Chaput, Mikael Fogelholm, Gang Hu, William D Johnson, Rebecca Kuriyan, Anura Kurpad, Estelle V Lambert, Carol Maher, José Maia, Victor Matsudo, Tim Olds, Vincent Onywera, Olga L Sarmiento, Martyn Standage, Mark S Tremblay, Catrine Tudor-Locke, Pei Zhao, Timothy S Church

**Affiliations:** 1 Pennington Biomedical Research Center, 6400 Perkins Road, Baton Rouge, LA 70808-4124, USA; 2 Children’s Hospital of Eastern Ontario Research Institute, Ottawa, Canada; 3 University of Helsinki, Helsinki, Finland; 4 St. Johns Research Institute, Bangalore, India; 5 UCT/MRC Research Unit for Exercise Science and Sports Medicine, Faculty of Health Sciences, University of Cape Town, Cape Town, South Africa; 6 School of Health Sciences / Sansom Institute, University of South Australia, Adelaide, Australia; 7 CIFI2D, Faculdade de Desporto, University of Porto, Porto, Portugal; 8 Centro de Estudos do Laboratório de Aptidão Física de São Caetano do Sul (CELAFISCS), Sao Paulo, Brazil; 9 Kenyatta University, Nairobi, Kenya; 10 Universidad de los Andes, Bogota, Colombia; 11 University of Bath, Bath, UK; 12 Tianjin Women’s and Children’s Health Center, Tianjin, China

**Keywords:** Adiposity, Behaviour, Pediatric obesity, Energy balance, Physical activity, Nutrition

## Abstract

**Background:**

The primary aim of the International Study of Childhood Obesity, Lifestyle and the Environment (ISCOLE) was to determine the relationships between lifestyle behaviours and obesity in a multi-national study of children, and to investigate the influence of higher-order characteristics such as behavioural settings, and the physical, social and policy environments, on the observed relationships within and between countries.

**Methods/design:**

The targeted sample included 6000 10-year old children from 12 countries in five major geographic regions of the world (Europe, Africa, the Americas, South-East Asia, and the Western Pacific). The protocol included procedures to collect data at the individual level (lifestyle, diet and physical activity questionnaires, accelerometry), family and neighborhood level (parental questionnaires), and the school environment (school administrator questionnaire and school audit tool). A standard study protocol was developed for implementation in all regions of the world. A rigorous system of training and certification of study personnel was developed and implemented, including web-based training modules and regional in-person training meetings.

**Discussion:**

The results of this study will provide a robust examination of the correlates of adiposity and obesity in children, focusing on both sides of the energy balance equation. The results will also provide important new information that will inform the development of lifestyle, environmental, and policy interventions to address and prevent childhood obesity that may be culturally adapted for implementation around the world. ISCOLE represents a multi-national collaboration among all world regions, and represents a global effort to increase research understanding, capacity and infrastructure in childhood obesity.

## Background

Childhood obesity is the result of a complex interaction of multiple behavioural, biological and environmental factors that adversely impact long term energy balance. The prevalence of childhood obesity remains high, and there are regional differences in both the level of obesity and rates of increase that have occurred over time [1,2]. Lifestyle behaviours such as physical activity and dietary intake have been associated with childhood obesity in many countries; however, the extent to which differences in lifestyle behaviours predict childhood obesity may not be the same across all regions of the world [3,4]. These behaviours are complex, and are affected by multiple levels of influence, including individual characteristics and higher-order factors such as local and national policies, the physical or built environment, numerous behavioural settings and domains, and local cultures [5,6]. The purpose of this paper is to describe the design and methods of the International Study of Childhood Obesity, Lifestyle and the Environment (ISCOLE) which will explore these issues.

Key questions about how lifestyle behaviours should be modified to address the problem of childhood obesity on a global stage can be best answered by a multi-national study in which both physical activity and dietary intake are measured in a standardized manner, and key measures of the multiple levels of influence are obtained in several countries. Each country and geographical jurisdiction has its own limited variability in the prevalence of obesity and each of the potentially modifying factors at different levels, yet an international study is able to maximize variability in these factors. The results will be important for adapting lifestyle- and policy-based childhood obesity interventions for implementation in different geographic regions and cultures.

To our knowledge, previous multi-country childhood obesity studies have focused on specific geographic regions (mainly Europe), rather than having a global representation of research sites that included both developing and developed countries. Further, few international studies of children and adolescents have included comprehensive, robust indicators of lifestyle behaviours (e.g. physical activity, food consumption, sedentary behaviour, sleep, etc.) and measures of higher-order characteristics and directly measured body mass or adiposity. However, there are several published and on-going studies that have informed the rationale and design of ISCOLE.

The Health Behaviour in School-Aged Children Study (HBSC) is currently conducted among 11, 13 and 15 year-old children in 43 countries (Europe, USA and Canada) [7]. Results from two previous cycles of the HBSC (2001–2002 and 2005–2006) indicate a consistent relationship between self-reported physical activity and overweight across countries; however, the relationship with dietary variables has been less consistent [3,4]. The Healthy Lifestyle in Europe by Nutrition in Adolescents (HELENA) study is a multi-country European collaboration that focuses on the health and health behaviour of adolescents [8]. Recent analyses from the HELENA study have examined associations among obesity and several lifestyle behaviours, including physical activity, sedentary behaviour, dietary habits, and sleep duration [9-11]. The European Youth Heart Study (EYHS) was a 4-country examination of cardiovascular risk factors and their related influences in 9 and 15 year-old children [12]. Results from the EYHS indicate a significant association between objectively measured physical activity (using accelerometers) and adiposity (sum of skinfolds), although the variance in adiposity explained by physical activity was <1% [13]. Further analyses incorporating self-reported television viewing habits suggest that physical activity and television viewing may have independent effects on adiposity and cardiovascular risk factors [14].

In addition to purely observational studies of lifestyle behaviours and obesity in children, several initiatives are under way to develop multi-national childhood obesity intervention consortia and studies [15]. For example, the European IDEFICS (identification and prevention of dietary- and lifestyle-induced health effects in children and infants) study was designed as an 8-country cohort study with an embedded intervention [16]. To date, baseline IDEFICS data have been used to explore associations of lifestyle behaviours such as sedentary behaviour [17], dietary preferences [18] and socioeconomic status [19] with obesity.

The existing international studies have demonstrated significant associations between lifestyle behaviours and obesity within the context of developed countries; however, the role of higher-order environmental correlates of lifestyle behaviours on adiposity and obesity are not well understood. The ENERGY (EuropeaN Energy balance Research to prevent excessive weight Gain among Youth) study was designed to explore personal family and school-level environmental correlates of energy balance-related lifestyle behaviours in 7 European countries [20]. However, there is a need to explore these multi-level relationships and obesogenic environments using both objective methods and self-report in a large multi-national sample of children from diverse cultural and socio-economic backgrounds. It is also important to have both high and low-medium income countries in the same study in order to get insight into cultural and socioeconomic aspects of environmental determinants of health behaviours and obesity in children. ISCOLE was established to address these issues, and the purpose of this paper is to present the design and methods of ISCOLE.

The primary aim of ISCOLE is to determine the relationships between lifestyle behaviours and obesity in a multi-national study of children, and to investigate the influence of higher-order characteristics such as behavioural settings, and the physical, social and policy environments, on the observed associations within and between countries. Our over-arching hypothesis is that the relationships between lifestyle behaviours and obesity will differ across countries and across different environmental settings. Much of the information available on the role of lifestyle behaviours and multiple levels of influence on childhood obesity has come from studies conducted in high income, developed countries, but studies from developing countries are limited. Thus, the results of ISCOLE will provide important new information that will inform the development of lifestyle and environmental interventions to address and prevent childhood obesity that can be adapted for implementation around the world.

## Methods/design

ISCOLE is a multi-national cross-sectional study conducted in 12 countries (Australia, Brazil, Canada, China, Colombia, Finland, India, Kenya, Portugal, South Africa, United Kingdom, United States) from five major geographic regions of the world (Europe, Africa, the Americas, South-East Asia and the Western Pacific) (Table 1).

**Table 1:** **International Study of Childhood Obesity**, **Lifestyle and the Environment (ISCOLE) field site characteristics**

**WHO region / country**	Human development index*	Gini Index (year)**	World bank classification***	**ISCOLE site location**	**Population size**^†^	**ISCOLE Site principal investigators**
Europe						
Finland	0.882 (Very High)	26.9 (2000)	High Income	Helsinki, Espoo & Vantaa	1,060,701	Mikael Fogelholm
United Kingdom	0.863 (Very High)	36.0 (1999)	High Income	Bath & North East Somerset	177,700	Martyn Standage
Portugal	0.809 (Very High)	38.5 (1997)	High Income	Porto	237,584	José Maia
Africa						
Kenya	0.509 (Low)	47.7 (2005)	Low Income	Nairobi	3,138,369	Vincent Onywera
						Mark Tremblay
South Africa	0.619 (Medium)	63.1 (2009)	Upper-Middle Income	Cape Town	3,497,097	Estelle V. Lambert
The Americas						
Canada	0.908 (Very High)	32.6 (2000)	High Income	Ottawa	883,391	Mark Tremblay
						Jean-Philippe Chaput
United States	0.910 (Very High)	40.8 (2000)	High Income	Baton Rouge	802,484	Catrine Tudor-Locke
Colombia	0.710 (High)	55.9 (2010)	Upper-Middle Income	Bogotá	7,674,366	Olga Sarmiento
Brazil	0.718 (High)	54.7 (2009)	Upper-Middle Income	São Paulo	19,889,559	Victor Matsudo
South-East Asia						
India	0.547 (Medium)	33.4 (2005)	Lower-Middle Income	Bangalore	9,588,910	Anura Kurpad
						Rebecca Kuriyan
Western Pacific						
China	0.687 (Medium)	42.6 (2002)	Upper-Middle Income	Tianjin	10,290,987	Pei Zhao
						Gang Hu
Australia	0.929 (Very High)	35.2 (1994)	High Income	Adelaide	1,212,982	Timothy Olds
						Carol Maher

*obtained from United Nations Development Programme. Human Development Report 2011. Sustainability and Equity: A Better Future for All. New York NY: Palgrave Macmillan; 2011; **obtained from World Bank (http://data.worldbank.org/indicator/SI.POV.GINI/); ***obtained from World Bank. World Development Indicators 2012. Washington, DC: The World Bank; 2012;^†^represents the population size of the city or general area where children were sampled.

**Figure 1:**
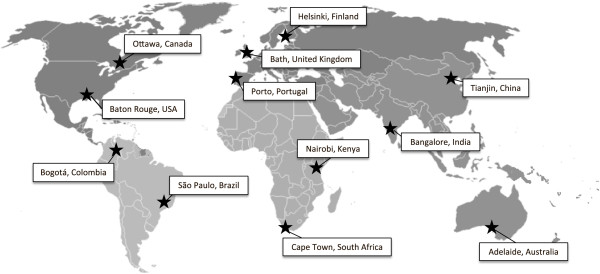
**Geographic distribution of the International Study of Childhood Obesity**, **lifestyle and the environment (ISCOLE) sites.**

Reflecting the variability in the ISCOLE sample, selected countries differed in several socio-economic indicators. According to World Bank classifications, ISCOLE countries span low income (Kenya), lower-middle income (India), upper-middle income (Brazil, China, Colombia, South Africa) and high-income economies (Australia, Canada, Finland, Portugal, USA, United Kingdom) [21]. Further, the ISCOLE countries also span a continuum of the Human Development Index (HDI), which is a composite score based on life expectancy, gross national income, literacy and school participation [22]. Accordingly, the countries were classified as low (Kenya), medium (China, South Africa and India), high (Brazil and Colombia) and very high HDI (Australia, United States, Canada, Finland, United Kingdom, Portugal) [22]. Additional contextual information can be obtained using the Gini index, which reflects the extent to which the distribution of income or consumption expenditure among individuals or households within an economy deviates from a perfectly equal distribution [23]. A Gini index of 0 represents perfect equality; whereas an index of 100 represents perfect inequality. Among ISCOLE countries, the Gini index ranges from 26.9 (Finland) to 63.1 (South Africa). Table 1 presents a detailed presentation of each of the 12 study sites’ rankings, population size, and principal investigators, and Figure 1 presents the geographic locations of the data collection sites. The descriptive socio-economic information provided in Table 1 is assessed at the country level (i.e. HDI, Gini index) and there is further variability within countries. The data collection sites span a range of north and south latitudes and the associated climatic and cultural differences.

### Study sample

The recruitment goal of ISCOLE was to enroll at least 500 children, gender balanced, with a mean age of 10 years from each of the 12 study sites. Given the anticipated difficulty in obtaining a school-based sample of children who would be exactly 10 years of age, each study site determined the best grade levels to target that would ensure a final sample with minimal variability around a mean age of 10 years. The sample necessarily included children aged 9–11 years of age.

Each study site identified one or more school districts (within reasonably close proximity to the local study center), to provide a sufficient population to draw a sample of 500 students. In an effort to maximize comparisons across ISCOLE sites, the sampling frame included students from urban and suburban areas, but not from rural areas. Rural areas were excluded from the sampling frame due to logistical concerns related to data collection raised by site investigators located in research institutes without access to rural populations. Further, the instruments used for measuring aspects of the neighborhood environment have not been adapted for use in rural areas.

Since within these general parameters, recruitment efforts necessarily differed between ISCOLE sites, Table 2 presents a short description of the sampling strategies employed. The primary sampling unit was schools and the secondary sampling unit was classes in the school that best corresponded to 10-year-old students. The primary sampling frame of schools was typically stratified by indicators of socio-economic status in order to maximize variability within sites. School-specific sampling frames were the lists of all classes corresponding to children on average 10 years of age.

**Table 2:** **Sampling methods employed by International Study of Childhood Obesity**, **Lifestyle and the Environment** (**ISCOLE**) **sites**

**ISCOLE site**	**Sampling strategy**
Australia	A stratified probability sampling frame was used, aiming to ensure that each 5^th^ grade child in the school system has an equal chance of being selected. Schools were initially stratified into tertiles based on the Index of Community Socio-Educational Advantage (ICSEA). Schools were randomly chosen from within each ICSEA tertile, with the probability of being chosen proportional to the estimated enrolment in 5^th^ grade. Once the required number of children was enrolled from a tertile (200 children), enrolment continued exclusively from the remaining tertiles.
Brazil	There is variability in socioeconomic status between schools in the region of Sao Caetano do Sul. Public schools represent the lower socio-economic strata, and private schools reflect the higher socio-economic strata. Random lists of public and private elementary schools in the region were generated, and schools were selected for each list at a ratio of 4 (public) to 1 (private). If a school refused to participate in the study, it was replaced by the school next on the list. Twenty schools were sampled (16 public and 4 private), and 5^th^ grade students continued to be sampled in each school in order to have between 25–30 students in each school.
Canada	Schools were drawn from the Ottawa Region. Schools were stratified into four groups with proportional representation (English Public, French Public, English Catholic, French Catholic). All schools within each stratum were invited to participate and the first to respond were included into the study until each stratum was filled. Children in 5^th^ grade were selected from the schools to participate.
China	Three regions (2 urban districts and 1 suburban district) of Tianjin city were selected and stratified according to three levels of socio-economic status. Within each stratum, 2 schools are selected randomly from a list of all public schools, with a total of 6 schools to participate. If the selected school refused to join the project, it was replaced by the next randomly selected school. Each school ensured an average sample size of 85–90 grade 4^th^ grade students.
Colombia	A list of public and private schools in Bogotá were selected according to the following inclusion criteria: 1) schools in urban area; 2) including boys and girls; 3) having a morning schedule; 4) enrolling students from elementary, middle and high-school; 5) belonging to January-December calendar, and 6) not being a school for a disabled population (blind, deaf, etc.). Given the distribution of students who attend public (76%) vs. private schools (24%), 15 public schools and 5 private schools were selected randomly. Schools were sorted into high SES, middle SES and low SES. The sample will result in a minimum recruitment of 600 4^th^, 5^th^ and 6^th^ grade children to obtain 500 children in 20 schools assuming an 83% response rate.
Finland	The sampling frame was a complete list of primary schools in the capital region (cities of Helsinki, Vantaa and Espoo, total population about 1 million or 19% of Finnish population). Schools were first stratified by city and then by area SES (high, low) based on socio-economic characteristics of their geographical location (educational level, if available, otherwise income level). From each of these 6 strata (city/SES), three to six schools were randomly selected to represent the distribution of pupils and SES within the total sampling area. A reserve list was used to account for school withdrawal. Children in 4^th^ grade were selected from the schools to participate.
India	Fee structures of different private schools catering to different socio-economic status were obtained. Based on this, a classification was made into low, middle and high socio-economic status. Three to four consenting schools were selected from each stratum. If a school declined the invitation to participate in the study, another school of the same fee structure was selected. The children from 5^th^ grade were sampled to have at least 60–70 students from each school.
Kenya	Non-boarding primary schools from Nairobi County were stratified into public and private (boarding schools were not sampled). The schools were then selected proportional to the distribution of public and private school attendance. Non-compliant schools were replaced with the next conveniently selected school from the group. Children in 5^th^ grade were selected from the schools to participate.
Portugal	There is little variability in socio-economic status at the school level in Porto; thus schools were randomly selected from a list provided by the North Regional Education Directory Board. If a non-compliant school was found, it was replaced by the next random school selected from the group. Twenty two schools were sampled, and from each, 5^th^ grade students were sampled in order to have 25–30 students in each school.
South Africa	The sampling frame was a list of all public schools within the geographic area of study eligibility. The list was stratified according to SES quintiles and at least 4 schools were randomly selected from each stratum for a total of at least 20 schools. If a school declined the invitation to participate in the study, another school of the same socio-economic quintile was randomly selected. Children in the 4^th^ and 5^th^ grades were selected from the schools to participate.
United Kingdom	The sampling frame was a complete list of primary schools in the Bath & North East Somerset and West Wiltshire regions. Schools were stratified according to size and socio-economic characteristics (e.g., free school meal entitlement). From each stratum, a proportional cluster was selected. Specifically, schools were randomly selected using the *probability proportional to size* approach and a reserve-list compiled to account for school withdrawal. Children in the 5^th^ and 6^th^ years were selected from the schools to participate.
United States	A complete list of public and private schools enrolling 4^th^ grade students in East Baton Rouge Parish was assembled. Private schools (collectively a stratum) were sampled separately. The public schools were sorted into quartiles (strata) according to% free and/or reduced lunch. Thus, there were five strata to sample from (4 public and 1 private). All schools were placed in random order within each stratum. Each school was approached according to the random order established within each stratum until a minimum of 4 schools were selected from each stratum, for a total minimum of 20 schools across strata, resulting in a minimum enrollment of 500 4^th^ grade children.

Given that the primary sampling strategy was based on schools, ISCOLE data collection was conducted during the school year, which varied across countries. When the school year spanned more than one season, the data collection period was split into phases to ensure that different seasons were covered. Data collection for ISCOLE began in September of 2011, and it is anticipated that it will be fully completed by December 2013. Data collection proceeded in a staggered fashion, such that each site completed their data collection over 12 months, or across one school year.

### Measurements

The ISCOLE protocol includes data collected from several objective and subjective sources, including the students, their parents, school administrators, and direct observations of the school environment by trained ISCOLE staff. Table 3 presents an overview of the measurements obtained in ISCOLE.

**Table 3:** **Summary overview of data collected in the International Study of Childhood Obesity**, **Lifestyle and the Environment** (**ISCOLE**)

	**Source**	**Measurements**
Obesity	Child Participant	Anthropometry
		- stature, body mass, arm circumference, waist circumference, sitting height
		- body mass index (BMI), sitting height/stature ratio, waist circumference/stature ratio
		Bioelectrical Impedance
		- impedance and body fat
Lifestyle	Child Participant	Physical Activity and Sedentary Behavior
		- accelerometry - minutes of moderate-to-vigorous physical activity (MVPA), sedentary time, steps/day
		- self-reported daily physical activity, outdoor time, television viewing and computer use, physical education, active transport to school, motivation for and attitudes towards physical activity
		Diet and Nutrition
		- food frequency questionnaire (FFQ), eating in front of television, frequency of eating breakfast, school lunches and outside of the home, emotional eating
		Other
		- sleep duration and quality (from both questionnaire and accelerometry), self-rated health and well-being
Demographics and Family Health	Parent/Guardian	- ethnicity of participant, socioeconomic status of family, residential address, health history of participant and parents, family structure, age, education, stature and weight of biological parents
Home and Neighborhood Environment	Parent/Guardian	- neighborhood social capital, home social environment, home and neighborhood food environment, home and neighborhood physical activity environment
School Environment	School Administrator	- school facilities, healthy eating and physical activity policies, extracurricular activities, frequency of physical education and breaks (recess), availability of healthy and unhealthy food
	School Audit	- support for active participation, sports and play amenities, aesthetics, food environment, competitive food environment

#### Anthropometry and bioelectrical impedance

A battery of anthropometric measurements was taken according to standardized procedures. Stature and sitting height were measured without shoes using a Seca 213 portable stadiometer (Hamburg, Germany), with the participant’s head in the Frankfort Plane. Stature was measured with participant fully erect, feet together, and at the end of a deep inhalation, and sitting height was measured while seated on a table with legs hanging freely and arms resting on the thighs [24]. Waist circumference was measured at the end of gentle expiration with a non-elastic tape held midway between the lower rib margin and the iliac crest [25]. Mid-upper-arm circumference was measured on the right arm using a non-elastic tape held midway between the acromion and olecranon processes, with arm hanging loosely at the side of the body [24]. Each measurement was repeated, and the average used for analysis (a third measurement was obtained if the first two measurements were greater than 0.5 cm apart and the average of the two closest measurements was used for analysis).

The participant’s body mass, impedance and percentage body fat were measured using a portable Tanita SC-240 Body Composition Analyzer (Arlington Heights, IL) after all outer clothing, heavy pocket items and shoes and socks were removed. Two measurements were obtained, and the average was used in analysis (a third measurement was obtained if the first two measurements were more than 0.5 kg or 2.0% apart, for body mass and percentage fat, respectively, and the closest two were averaged for analysis). In addition, impedance values were recorded for future analyses. Due to harsh field conditions in some sites, a back-up scale was carried by the assessment team to ensure that body weight was obtained.

The Body Mass Index (BMI; body mass (kg)/height (m^2^)), waist-to-stature ratio, and sitting height-to-stature ratio were calculated.

#### Demographics and family health history

A demographic and family health history questionnaire was completed by parents (see Additional file 1: ISCOLE Demographic and Family Health Questionnaire). The questionnaire collected information on basic demographics, ethnicity, family health and socioeconomic factors.

#### Diet and lifestyle information

A diet and lifestyle questionnaire that included questions related to food consumption, physical activity, sedentary behaviour, sleep, health and well-being was administered to all ISCOLE student participants (see Additional file 2: ISCOLE Diet and Lifestyle Questionnaire). This questionnaire is a compilation of validated items obtained from several different sources (described below) as well as new questions designed by ISCOLE investigators where no suitable previous alternatives were found. Technicians were trained to administer the questionnaire in a standardized fashion in order to minimize bias, and provisions were made to administer the questionnaire via an interview for participants with low levels of literacy. Questionnaires were checked for completeness at the time of data collection in order to ensure high quality data.

A food frequency questionnaire (FFQ) that was adapted from the HBSC Study [26] was integrated into the diet and lifestyle questionnaire. The FFQ asks the child about several different types of food consumed in a “usual” week. The FFQ lists 23 food categories and has examples of individual food items, but no portion sizes. The food items included in the FFQ were standardized as much as possible across ISCOLE sites; however, regional variation in food consumption patterns necessitated some cultural and regional adaptations of some of the items and examples provided. In order to better understand the interactions between television viewing and dietary intake, a brief 5-item FFQ was adapted from a published study that asks the children about the consumption of different types of snacks while viewing television [27]. Additional questions related to the consumption of breakfast [26], school lunches [28], and the number of meals prepared or eaten away from home were also included.

Several physical activity and sedentary behaviour questions were obtained from the U.S. Youth Risk Behavior Surveillance System [29]. These questions related to obtaining adequate amounts of physical activity, the amount of time spent watching television or playing video games, and the number of physical education classes attended per week. Questions related to active transport to school were adapted from the Canadian component of the 2009/10 HBSC Study [30]. In order to capture potential windows of opportunity for physical activity, several questions were also developed by the ISCOLE investigators to probe the amount of time the children spent outside before school, after school, and on the weekend.

There is emerging evidence of a link between sleep and the development of obesity [31-33]. Therefore, several questions related to sleep patterns, duration and quality were developed by the ISCOLE investigators for inclusion in the diet and lifestyle questionnaire. The questionnaire also contained sections to measure psychological constructs related to physical activity and dietary behaviour, including adapted scales for motivation for physical activity [34], self-efficacy for physical activity [35], and emotional eating [36]. Finally, questions on health-related quality of life were included, derived from the validated Kidscreen-10 quality of life scale [36].

#### Accelerometry

The Actigraph GT3X + accelerometer (ActiGraph, Pensacola, FL) was used to objectively monitor physical activity, sedentary behaviour and sleep time. The accelerometer was worn at the waist on an elasticized belt, on the right mid-axillary line. Since other studies have reported problems with achieving minimal wearing time with a waking hours protocol (i.e., sanctioning daily instrument removal), ISCOLE children were encouraged to wear the accelerometer 24 hours per day for at least 7 days (plus an initial familiarization day and the morning of the final day), including 2 weekend days. The minimal amount of accelerometer data that was considered acceptable was 4 days with at least 10 hours of wear time per day, including at least one weekend day. Following the final day of data collection, accelerometers were returned to the study site, and the research team verified the data for completeness using the most recent version of the ActiLife software (version 5.6 or higher; ActiGraph, Pensacola, FL) available at the time. Local study sites could ask children to wear the accelerometer for additional days (to a maximum of 14 days) to ensure that the minimal data requirements were met.

#### Assessment of biological maturity

Although all student participants in ISCOLE were 9–11 years of age, they differed in their stage of biological maturity. Several methods exist to assess biological maturity, including the assessment of secondary sex characteristics, skeletal age, dental maturation, or somatic maturation [37]. All options were considered for use in ISCOLE; however, the method that was deemed most feasible across all countries was to use estimates of somatic maturation. Two methods are employed: 1) percentage of predicted adult stature attained, using the Khamis-Roche method [38] to predict adult stature, and 2) the maturity offset [39].

The rationale for using percentage of adult stature attained is that two children of the same age can be the same stature, but one may be closer to their final adult stature, and hence is more advanced in somatic maturation. Given that the final adult stature of the children was not known, it was predicted using their chronological age, stature, body mass and mid-parent stature (average of father’s and mother’s stature) [38]. Another important indicator of somatic maturation is age at peak height velocity, which is typically assessed from serial measurements of the child throughout adolescence. Age at peak height velocity is a commonly used indicator of somatic maturity [37]. Given that ISCOLE is a cross-sectional study, the method of Mirwald and colleagues [39] was used to predict years from peak height velocity, or the “maturity offset” from age, sex, sitting height, stature and body mass.

#### Neighborhood and home environment

A neighborhood and home environment questionnaire was completed by the parents (see Additional file 3: ISCOLE Neighborhood and Home Environment Questionnaire). The questionnaire was adapted from the Neighborhood Impact on Kids (NIK) study survey [40] which drew on questions from other validated instruments [41-43]. The questionnaire includes items related to neighborhood social capital (collective efficacy and the degree to which persons in the neighborhood know each other and engage socially), the home social environment (parental support for and modeling of physical activity), the home and neighborhood food environments (availability of various healthy or unhealthy food and drink choices in the home, and the availability and types of food stores in the neighborhood) and the home and neighborhood physical activity environment and neighborhood built environment (availability of electronics for the child’s personal use, access to and use of play equipment in the home environment, access to and use of various places where a child can be physically active, and the suitability of the neighborhood environment for walking and physical activity).

#### School environment

The school environment was assessed using a questionnaire completed by a school administrator (e.g. principal)(see Additional file 4: ISCOLE School Environment Questionnaire), and a direct audit of the school environment, performed by ISCOLE staff. The school administrator questionnaire was adapted from the healthy eating and physical activity modules of the Healthy School Planner [44] used in the Canadian School Health Action, Planning and Evaluation System (SHAPES) [45]. The questionnaire includes items related to school facilities, healthy eating and physical activity policies, extracurricular activities, frequency of physical education and breaks (recess), promotion of active transportation, and the availability of healthy and unhealthy foods in the cafeteria and vending machines. In addition, questions regarding food items sold as part of fundraising efforts were incorporated from the U.S. School Health Policies and Practices Study (SHPPS) [46]. Two additional questions were added by ISCOLE investigators to capture the number of days that students attend school during the academic year and the amount of class time mandated for physical education.

Specially trained ISCOLE study staff completed an environmental audit of each participating school. A single data collector performed each school audit. For at least 5% of the schools within a site, another similarly trained data collector performed a second, independent audit in order to determine item reliability and to monitor consistency in adhering to item definitions. The school audit tool was used to collect directly-observed information pertaining to the school built and food environments. Components of the audit tool addressing the school built environment were taken from the school audit tool used in the SPEEDY (Sport, Physical activity and Eating behaviour: Environmental Determinants in Young people) study [47,48], which has acceptable reliability (agreement between pairs of auditors range from 76% to 99% across components) and good construct validity (components of the tool were able to discriminate physical activity levels of children attending schools in the highest and lowest quintiles) [48]. A question regarding whether fast food restaurants were visible from any of the school entrances was added by ISCOLE investigators in order to better characterize the food environment of the area surrounding the school. Components of the audit tool addressing the *à la carte* school food environment were modified based on that used in the IDEA (Identifying Determinants of Eating and Activity) study [49]. The wording of choices, including examples of environmental features (e.g. “sidewalk” vs “footpath”) and customary food items, were adapted as necessary across ISCOLE sites. A photo dictionary was created to standardize data collection and capture unique features of the different countries.

### Ethical issues

The overarching ISCOLE protocol was approved by the Pennington Biomedical Research Center Institutional Review Board. Each adapted site-specific protocol was also approved by the ethical review boards at the participating institutions. Informed parent consent/ child assent forms and all questionnaires were translated and back-translated, as necessary, into the local language of each study site following approved procedures of the local institutions.

### Governance and study management

**Figure 2:**
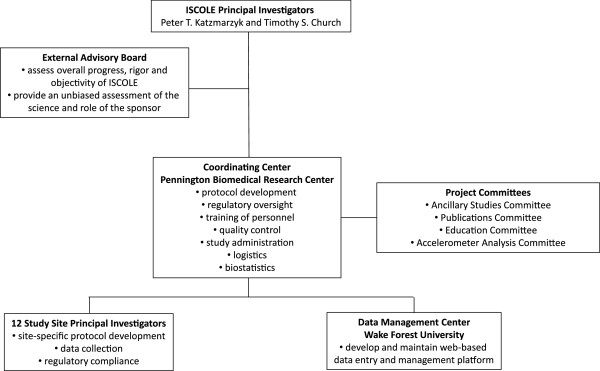
**Overview of governance and management of the International Study of Childhood Obesity**, **Lifestyle and the Environment (ISCOLE).**

A schematic describing the governance and management of ISCOLE is provided in Figure 2. The principal investigators were responsible for overseeing all aspects of the study. The role of the External Advisory Board was to assess the overall progress, rigor and objectivity of the study and to make recommendations to the management team regarding logistical issues, recruitment, safety issues, guidance on overall study direction and future goals. The members of the External Advisory Board were selected to be at arm’s-length from both the funder and the investigators in order that they could provide an unbiased assessment of the science and the role of the funder. The ISCOLE Coordinating Center in Baton Rouge, LA was responsible for the overall administration the study. Each of the ISCOLE study sites was overseen by a local site principal investigator, who was responsible for the all aspects of study implementation at the local level. With the exception of requiring that the study be global in nature, the study sponsor had no role in study design, data collection and analysis, decision to publish, or preparation of manuscripts.

### Data management

Given that the 12 data collection sites for ISCOLE were spread across all regions of the world presented some logistical challenges for data acquisition. To facilitate data collection, entry and management, a secure web-based system was developed by the Coordinating Center and the Research Information Systems group at Wake Forest School of Medicine (Winston-Salem, NC). The web-based data management system facilitated the flow of information and increased the level of communication within ISCOLE. Large volumes of data were entered remotely at each study site using a standard web-browser, and the system allowed both the study site staff and the Coordinating Center to monitor progress and produce missing data reports in real time.

### Training of personnel and quality control

Regional training sessions organized for all site-PIs and key staff were conducted by the ISCOLE Coordinating Center (Pennington Biomedical Research Center) in advance of data collection at each study site. All staff members were certified by trained experts as competent to make the required measurements, which involved the completion of on-line training modules, viewing protocol videos and successfully completing on-line exams prior to the hands-on training sessions and one-on-one training by experts.

**Figure 3:**
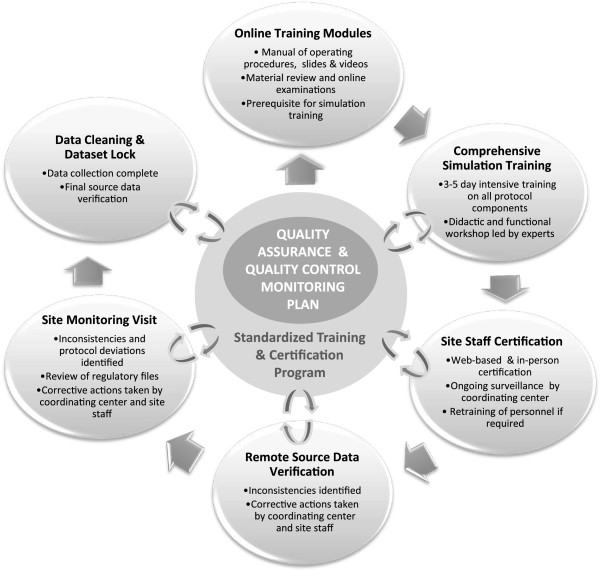
**Summary of the International Study of Childhood Obesity**, **Lifestyle and the Environment (ISCOLE) quality assurance**, **quality control and training program.**

Quality control and the rigorous standardization of measurement protocols across sites are critical to the success of a multi-center study. Quality control was a shared responsibility of all ISCOLE investigators. Figure 3 provides an overview of the training and quality control program of ISCOLE. The Coordinating Center at the Pennington Biomedical Research Center was responsible for coordinating protocol development and implementation at each site, overseeing the training and certification of study personnel, ensuring regulatory compliance, and monitoring data collection and remote data entry. Site principal investigators were responsible for regulatory compliance, ensuring that the standard protocol was implemented at their site, supervising and ensuring the accuracy of data entry, retraining study personnel as necessary, and maintaining all required regulatory documents in a binder. During data entry, key variables were checked for accuracy with assigned range checks. A review was required for any data entered outside of preset ranges. Through communication with the study sites, the Coordinating Center reconciled any questionable responses within 30 days.

Quality control was monitored during data collection through remote source document verification, monitoring data entry errors, and study site visits by Coordinating Center staff using a standard site-monitoring protocol. During site visits, regulatory binders were inspected to ensure regulatory compliance, an independent school audit was conducted by visiting Coordinating Center staff, and local study staff members were observed onsite performing data collection to ensure protocol adherence and proper documentation of measurements. Additional training was provided when necessary to maintain quality control. Data entry error rates, nature of the errors, and any quality control issues found during source data verification were discussed with the site staff and principal investigators. Any deficiencies identified were immediately corrected, with an option to stop enrollment until major deficiencies were corrected.

### Statistical analysis approach

Data on participant attributes such as demographic, anthropometric, and lifestyle behaviours will be summarized separately for boys and girls, within and across all study sites, as counts and percentages for categorical variables and means and standard deviations for continuous variables. Given that the primary aim of ISCOLE is to predict obesity as a function of lifestyle behaviours and environmental characteristics, general linear and nonlinear statistical models, including covariate-adjusted models, will be employed to investigate relationships between adiposity and its potential determinants. Multilevel random-effects models that treat schools within site and children within schools as well as schools within countries as random effects will be used for all analyses. Statistical significance will be defined as p < 0.05 with appropriate adjustments for multi-testing.

### Sample size calculations

In order to estimate sample size requirements, a regression model was developed to predict BMI from objectively monitored physical activity (accelerometer-determined activity counts/day) and self-reported caloric consumption (kcal/day) among 10-year olds in the 2005/2006 U.S. National Health and Nutrition Examination Survey. Based on estimates gleaned from the regression model, the sample size was determined under the following assumptions: 1) 10-year old students would be sampled in school clusters with an average of 25 students per school, 2) the 5% significance level was desirable, and 3) at least 90% power was required to detect as significant a predictor that explains 3% of the variability in BMI. It was determined that a site-specific minimum of 350 students would achieve a minimum power of 91%. Because the students were to be sampled in clusters by schools, a design effect of 1.3, estimated from Williamson et al. [50], was applied to produce a required sample size of 455. To further allow for students who may fail to provide valid and /or complete data, the enrollment target for each site was set at 500 students, within a target of at least 20 schools. The total sample size of approximately 6000 children across twelve countries should provide excellent power for detecting meaningful significant contributors to the hypothesized multi-level model.

## Discussion

ISCOLE is a multi-national collaborative study of lifestyle behaviours, the environment and obesity in children from around the world. This study extends the results from single-site and regional multi-center studies by incorporating local study research sites and collaborators from a broad spectrum of the world’s regions. The standardized protocol and rigorous training and quality control program will ensure accurate and reliable data, and will also lead to the further development of research capacity on energy balance among all participating institutions and countries. Further, the resulting dataset will be a valuable resource for the training and education of students and developing scientists from around the world well into the future, as well as for potential follow-up of participants to determine patterns of weight change.

The strengths and weaknesses of ISCOLE warrant discussion. The major strengths of the study include the recruitment of a large multi-national sample of children from low to high income countries across several regions of the world, the highly standardized measurement protocol, the use of direct measurements whenever possible, the rigorous quality control program, and the consideration of higher-order environmental characteristics. ISCOLE is currently a cross-sectional study with all of the inherent limitations of this design; however, the potential exists to follow the participants longitudinally in several countries. ISCOLE was not designed to provide representative data from each participating country or region; rather, the sampling strategy was designed to maximize variation in socio-economic status at each site. The ISCOLE countries span a wide range of health, social and economic indicators (see Table 1); however, the results of the study may not be directly generalizable to other countries. It should be noted that some of the measures used in ISCOLE are still rather crude, as is usual in epidemiological settings. For instance, a more profound assessment of the children’s diets would have been an advantage. However, assessing usual dietary intake in quantitative terms would have required considerably more resources, extensive individual participant burden, and more training for research personnel.

In summary, the results obtained in ISCOLE have the potential to increase our understanding of the correlates of childhood obesity and inform the development of lifestyle and environmental interventions designed to address and prevent childhood obesity that can be culturally adapted for implementation around the world. Diverse international evidence is needed to recommend environment and policy changes related to improving children’s lifestyles and reducing the prevalence of obesity. Cross-cultural comparisons may provide unique insight for future interventions.

## Abbreviations

BMI: Body mass index; ENERGY: EuropeaN energy balance research to prevent excessive weight gain among youth; EYHS: European youth heart study; FFQ: Food frequency questionnaire; HBSC: Health behaviours in school-aged children; HDI: Human development index; HELENA: Healthy lifestyle in Europe by nutrition in adolescents; IDEA: Identifying determinants of eating and activity; IDEFICS: Identification and prevention of dietary- and lifestyle-induced health effects in children and infants; ISCOLE: International study of childhood obesity, lifestyle and the environment; NIK: Neighborhood impact on kids; SHAPES: School health action, planning and evaluation system; SHPPS: School health policies and practices study; SPEEDY: Sport, physical activity and eating behaviour: environmental determinants in young people.

## Competing interests

The authors declare that they have no competing interests.

## Authors’ contributions

PTK researched the literature and drafted the manuscript. All authors contributed to the study design, critically reviewed the manuscript and approved the final version.

## Pre-publication history

The pre-publication history for this paper can be accessed here:

http://www.biomedcentral.com/1471-2458/13/900/prepub

## Supplementary Material

Additional file 1 ISCOLE Demographic and Family Health Questionnaire. Click here for file

Additional file 2 ISCOLE Diet and Lifestyle Questionnaire. Click here for file

Additional file 3 ISCOLE Neighborhood and Home Environment Questionnaire. Click here for file

Additional file 4 ISCOLE School Environment Questionnaire. Click here for file
