# Supplementary material for: The International Study of Childhood Obesity, Lifestyle and the Environment (ISCOLE): design and methods
Source: BMC Public Health. 2013 Sep 30;13:900. doi: 10.1186/1471-2458-13-900 (PMC3849927; doi:10.1186/1471-2458-13-900)
Supplement: Additional file 3 — ISCOLE Neighborhood and Home Environment Questionnaire. [file 1471-2458-13-900-S3.pdf]

ISCOLE NEIGHBORHOOD & HOME ENVIRONMENT QUESTIONNAIRE

Where "child" is mentioned, please respond only about the child who is participating in this study. Be as accurate as you can. There are no right or wrong answers. All information is strictly confidential.

A. NEIGHBORHOOD COHESION

**Do you agree or disagree with the following statements?**	Strongly disagree	Somewhat disagree	Neutral	Somewhat agree	Strongly agree
1. People around my neighborhood are willing to help their neighbors.	○	○	○	○	○
2. This is a close-knit neighborhood.	○	○	○	○	○
3. People in my neighborhood can be trusted.	○	○	○	○	○
4. People in my neighborhood generally don't get along with each other.	○	○	○	○	○
5. People in my neighborhood do not share the same values, attitudes or beliefs.	○	○	○	○	○

B. NEIGHBORS AND FRIENDS

1. Think about the neighborhood or area in which you live. In general, how well do you feel you know your neighbors?

Not at all	Just a little	Moderately well	Extremely well
○	○	○	○

2. About how often do you talk to or visit with your immediate neighbors (people in the 10-20 households that live closest to you)?

Never	Once a year or less	Several times a year	Once a month	Several times a month	Several times a week	Almost every day
○	○	○	○	○	○	○

C. NEIGHBORHOOD RESPONSE

**For the following statements, please mark how likely a neighbor would respond to, or take action in the following situations:**	Very unlikely	Unlikely	Neither likely nor unlikely	Likely	Very likely
1. If a group of neighborhood children were skipping school and hanging out on a street corner, how likely is it that your neighbors would do something about it?	○	○	○	○	○
2. If some children were spray-painting graffiti on a local building, how likely is it that your neighbors would do something about it?	○	○	○	○	○
3. If a child was showing disrespect to an adult, how likely is it that people in your neighborhood would scold that child?	○	○	○	○	○
4. If there was a fight in front of your house and someone was being beaten or threatened, how likely is it that your neighbors would break it up?	○	○	○	○	○
5. Suppose that because of budget cuts the fire station closest to your home was going to be closed down by the city. How likely is it that neighborhood residents would organize to try to do something to keep the fire station open?	○	○	○	○	○

D. FOODS IN THE HOME

**How often are the following foods/drinks available in your home?**	Never	Rarely	Sometimes	Often	Always
1. Chocolate candy	○	○	○	○	○
2. Other candy	○	○	○	○	○
3. Raw fruit (e.g., apples, oranges)	○	○	○	○	○
4. Cakes, brownies, muffins or cookies	○	○	○	○	○
5. Regular chips or crackers	○	○	○	○	○
6. Baked chips, low-fat crackers, pretzels	○	○	○	○	○
7. Raw vegetables (e.g., carrots)	○	○	○	○	○
8. 100% fruit juice	○	○	○	○	○
9. Juice drinks (e.g., Sunny delight)	○	○	○	○	○
10. Regular sodas with sugar	○	○	○	○	○
11. Diet or sugar free sodas	○	○	○	○	○
12. Sports drinks (e.g., Gatorade)	○	○	○	○	○
13. Fruit roll-ups or other dried fruit	○	○	○	○	○
14. Regular or 2% milk	○	○	○	○	○
15. 1% or fat-free milk	○	○	○	○	○
16. Sweetened breakfast cereal	○	○	○	○	○
17. Unsweetened breakfast cereal	○	○	○	○	○

E. WHERE YOU SHOP

**When you, or the main food shopper in your home, go food shopping, how often do you go to each of these types of stores?**	Never	Rarely	Sometimes	Often	Always
1. Large supermarket or discount warehouse	○	○	○	○	○
2. Small to medium food store	○	○	○	○	○
3. Convenience store	○	○	○	○	○
4. Farmer's market/produce stand	○	○	○	○	○
5. Other, specify: ____________________________	○	○	○	○	○

F. STORE ACCESS

**Please indicate whether the following statements are true of the store <u>where you usually buy groceries</u>.**	Yes	No	Not applicable
1. Close to location of my employment	○	○	○
2. Close to my child's school	○	○	○
3. Close to my home	○	○	○

G. FOOD SHOPPING

**The following questions apply to the store <u>where you usually buy groceries</u>.**	Strongly disagree	Somewhat disagree	Neutral	Somewhat agree	Strongly agree
1. Low-fat foods cost too much.	○	○	○	○	○
2. There is a large selection of fresh fruits and vegetables.	○	○	○	○	○
3. There is a large selection of low-fat products available.	○	○	○	○	○
4. The condition of fresh fruits and vegetables is poor.	○	○	○	○	○
5. Fruits and vegetables cost too much.	○	○	○	○	○

H. YOUR CHILD'S ELECTRONICS

Please indicate whether the following are <u>in your child's bedroom</u>.	Yes	No
1. TV	O	O
2. Computer	O	O
3. Video game system (non-hand held; Playstation, Xbox, etc.)	O	O

**Does your child have the following items for his/her own use?**	Yes	No
4. Cell phone or 2-way radio	O	O
5. Hand-held videogame players (Game Boy, Sony PSP, etc.)	O	O
6. Music systems (Ipod, stereo, radio, etc.)	O	O

For the next two questions, please think about your child's activities over the *past year*.

I. PLAY EQUIPMENT

**How often during the past year has your child used these items at or around home (or in a common apartment area)?**	Not available (Don't have)	Available but never use	Once a month or less	Once every other week	Once a week or more
1. Bike	O	O	O	O	O
2. Basketball hoop	O	O	O	O	O
3. Jump rope	O	O	O	O	O
4. Active video games (e.g., with dance pad, Wii, etc)	O	O	O	O	O
5. Sports equipment (like ball, racquets, bats, sticks)	O	O	O	O	O
6. Swimming pool	O	O	O	O	O
7. Roller skates, skateboard, scooter	O	O	O	O	O
8. Fixed play equipment (e.g., swing set, playhouse, jungle gym)	O	O	O	O	O

J. PLACES FOR YOUR CHILD'S PHYSICAL ACTIVITY

**How often during the past year has your child been <u>physically active</u> (including active play) in the following places?**	Never	Once a month or less	Once every other week	Once a week	2 or 3 times/ week	4 times/ week or more
1. Inside your home	O	O	O	O	O	O
2. In your yard or common area or in your driveway	O	O	O	O	O	O
3. At a neighbor's house, yard, or driveway	O	O	O	O	O	O
4. In a local street, sidewalk, or vacant lot/field	O	O	O	O	O	O
5. Indoor recreation or exercise facility (public or private; e.g., YMCA/Boys & Girls Club)	O	O	O	O	O	O
6. Beach, lake, river, or creek	O	O	O	O	O	O
7. Bike/hiking/walking trails, paths	O	O	O	O	O	O
8. Basketball court	O	O	O	O	O	O
9. Other playing fields/courts (like football, softball, tennis)	O	O	O	O	O	O
10. Small public park or playground	O	O	O	O	O	O
11. Large public park	O	O	O	O	O	O
12. Public open space that is not a park	O	O	O	O	O	O
13. School grounds (during non-school hours)	O	O	O	O	O	O

K. GETTING AROUND IN YOUR NEIGHBORHOOD

Please select the answer that best applies to you and your neighborhood. Within walking distance means within a 10-15 minute walk from your home.

	Strongly disagree	Somewhat disagree	Somewhat agree	Strongly agree
1. There are shops, stores, markets, and places to buy things I need within easy walking distance of my home/house.	○	○	○	○
2. There is a bus, subway, or train stop within walking distance from my home.	○	○	○	○
3. There are sidewalks on most streets.	○	○	○	○
4. There are NOT many dead end streets.	○	○	○	○
5. There are many different routes for getting from place to place.	○	○	○	○
6. There is a high crime rate.	○	○	○	○
7. The speed of traffic on most streets is usually slow (30 mph or less).	○	○	○	○
8. Most drivers go faster than the posted speed limits.	○	○	○	○
9. There are many interesting things to look at while walking in my neighborhood.	○	○	○	○
10. The traffic makes it difficult or unpleasant for my child to walk.	○	○	○	○
11. Streets have good lighting at night.	○	○	○	○
12. There are crosswalks and signals on busy streets.	○	○	○	○
13. There are many places to go within easy walking distance of my home.	○	○	○	○
14. I'm afraid of my child being taken or hurt by a stranger on local streets.	○	○	○	○
15. I'm afraid of my child being taken or hurt by a stranger in my yard, driveway, or common area.	○	○	○	○
16. I'm afraid of my child being taken or hurt by a stranger in a local park.	○	○	○	○
17. I'm afraid of my child being taken or hurt by a known "bad" person (adult or child) in my neighborhood.	○	○	○	○

L. DISTANCE TO LOCATIONS

About how long would it take <u>you</u> to walk from your home to the nearest places listed below? Please select the time it would take you to walk to each place, regardless of whether you/your child go there.

	1-5 min	6-10 min	11-20 min	21-30 min	31+ min	Don't know
1. Convenience/corner store/small grocery store/bodega	○	○	○	○	○	○
2. Supermarket	○	○	○	○	○	○
3. Fast food restaurant	○	○	○	○	○	○
4. Non-fast food restaurant	○	○	○	○	○	○
5. Indoor recreation or exercise facility (public or private; e.g., YMCA/Boys & Girls Club)	○	○	○	○	○	○
6. Beach, lake, river, or creek	○	○	○	○	○	○
7. Bike/hiking/walking trails, paths	○	○	○	○	○	○
8. Basketball court (including half-court)	○	○	○	○	○	○
9. Other playing fields/courts (like soccer, football, softball, tennis, skate park, etc.)	○	○	○	○	○	○
10. Small public park	○	○	○	○	○	○
11. Large public park	○	○	○	○	○	○
12. Public playground with equipment	○	○	○	○	○	○
13. School with recreation facilities open to the public	○	○	○	○	○	○

M. FAMILY

During a typical week, how often do you or another adult in the household:

	Never	1-2 days	3-4 days	5-6 days	Every day
1. Watch your child participate in physical activity or sports	○	○	○	○	○
2. Encourage your child to do sports or physical activity	○	○	○	○	○
3. Provide transport to a place where your child can do physical activity or play sports	○	○	○	○	○
4. Do a physical activity or play sports with your child	○	○	○	○	○
