# Supplementary material for: The International Study of Childhood Obesity, Lifestyle and the Environment (ISCOLE): design and methods
Source: BMC Public Health. 2013 Sep 30;13:900. doi: 10.1186/1471-2458-13-900 (PMC3849927; doi:10.1186/1471-2458-13-900)
Supplement: Additional file 4 — ISCOLE School Environment Questionnaire. [file 1471-2458-13-900-S4.pdf]

ISCOLE SCHOOL ENVIRONMENT QUESTIONNAIRE

A. SCHOOL CHARACTERISTICS

1. **What is your position at this school?** ☐ Principal ☐ Vice Principal ☐ Teacher ☐ Other:____________

2. **What is the total number of students in your school?** (Please estimate) _____ students

3. **What is the total number of teachers (full time equivalents) in your school?** (Please estimate) _____ teachers

4. **What grades are taught at your school?** _____ to _____

5. **How many days (excluding holidays) do your students attend school during the academic school year?** _______

B. POLICIES AND PRACTICES

For the following section, "policies" refers to any mandates issued by the state, the local school board, or any other agency, including policies developed by your school or (district/diocese), that affects your school environment and that have been officially adopted by your school or district. This section also asks about practices (what your students and staff are allowed to do on a regular basis) that you might follow to promote the health and well-being of students.

6. **Does your school have written policies or practices concerning physical activity?**

- ☐ **Yes**, existing written policies
- ☐ **Yes**, written policies still under development
- ☐ **Yes,** practices
- ☐ No
- ☐ N/A

7. **Does your school have written policies or practices concerning healthy eating?**

- ☐ **Yes**, existing written policies
- ☐ **Yes**, written policies still under development
- ☐ **Yes,** practices
- ☐ No
- ☐ N/A

8. **Does your school have a committee that oversees or offers guidance on the development of policies and practices concerning physical activity and healthy eating at your school (e.g., health action team, school health or wellness council)?**

- ☐ **Yes**, both physical activity and healthy eating
- ☐ **Yes**, physical activity only
- ☐ **Yes,** healthy eating only
- ☐ No

C. PHYSICAL ACTIVITY

9. What percent of students participate in the following extracurricular activities offered by your school? (Please estimate)

		Not available	*Less than 10%*	*10-24%*	*25-49%*	*50%+*
a.	Varsity/interschool athletics	☐	☐	☐	☐	☐
b.	Intramural athletics or physical activity clubs (including dance)	☐	☐	☐	☐	☐
c.	Academic/hobby clubs (e.g., chess, astronomy)	☐	☐	☐	☐	☐
d.	Arts-based clubs (e.g., drama, music, photography)	☐	☐	☐	☐	☐

10. Does your school offer late bus/transportation service to students who participate in extra-curricular activities?

Yes ☐ No ☐

11. From the following list, please indicate which sports are offered in your interschool or intramural athletics programs available to students in grade 4:

☐ a. Not applicable, school does not offer interschool or intramural athletics to students in grade 4

	Varsity/ Interschool	*Intramural*		*Varsity/ Interschool*	*Intramural*
b. Basketball	☐	☐	j. Gymnastics	☐	☐
c. Volleyball	☐	☐	k. Wrestling	☐	☐
d. Soccer	☐	☐	l. Track & Field	☐	☐
e. Football	☐	☐	m. Badminton	☐	☐
f. Baseball/softball	☐	☐	n. Swimming	☐	☐
g. Rugby	☐	☐	o. Skiing	☐	☐
h. Ice Hockey	☐	☐	p. Ultimate Frisbee	☐	☐
i. Lacrosse	☐	☐	q. Other	☐	☐

For the following questions, please consider students in grade 4 when answering.

12. How many breaks of 15 to 29 minutes do students in grade 4 have in a day?

☐ zero ☐ 1 ☐ 2 ☐ 3 or more

13. How many breaks of 30 minutes or more do students in grade 4 have in a day?

☐ zero ☐ 1 ☐ 2 ☐ 3 or more

14. How much class time is mandated by your [Province/Territory/State…] to be allotted to physical education (PE)/Daily Physical Activity (DPA) for students in grade 4?

______ **minutes per** *[check the box indicating the time unit]* **week** ☐ **day** ☐

☐ No specific amount is mandated

15. Compared to the class time allotted to physical education (PE)/Daily Physical Activity (DPA) for grade 4 as mandated by your [Province/Territory/State…], do students in grade 4 in your school receive on average:

- ☐ Less than the mandated amount
- ☐ Approximately the mandated amount
- ☐ More than the mandated amount
- ☐ No specific amount is mandated

16. To the best of your knowledge, how well do each of the following statements characterize your school?

	A lot	*Some*	*Very little*	*Not at all*	*Don't know*
a. We use physical activity as a reward	☐	☐	☐	☐	☐
b. We promote physical activity during or as part of special events	☐	☐	☐	☐	☐
c. We integrate physical activity into other curriculum areas	☐	☐	☐	☐	☐
d. We use physical activity as a punishment for bad behavior (e.g., withholding recess, administering push-ups or laps).	☐	☐	☐	☐	☐

17. Does your school promote *active transportation* to and from school in any of the following ways?

	Yes	*No*	*Don't know*
a. Identify safe routes to use for walking and cycling to and from school (e.g., with signs, in newsletters, etc.)	☐	☐	☐
b. Provide crossing guards at intersections to encourage safe walk-to-school routes	☐	☐	☐
c. Designate a 'car free zone' to provide safe walking areas around the school	☐	☐	☐
d. Allow students to bring bicycles on school property	☐	☐	☐
e. Allow students to bring small wheel vehicles (e.g., rollerblades, scooters, skateboards) on school property	☐	☐	☐
f. Encourage the use of helmets and safety gear for those who use bicycles and small wheel vehicles to get to school	☐	☐	☐
g. Organize occasional 'walk to school days' , walking clubs, or programs like 'walking school buses' (where parents or older students walk around the neighborhood and pick up walkers at designated points)	☐	☐	☐

D. SCHOOL FACILITIES

18. Do the majority of students at your school have regular access to any of the following during school hours*?
*During school hours means from the first bell to the last bell, including both instructional and non-instructional time (e.g., lunch).

		Yes, on grounds only	*Yes, off grounds only*	*Yes, both on and off grounds*	*No*	*Don't know*
a.	Gymnasium	☐	☐	☐	☐	☐
b.	Other large room suitable for physical activity (e.g., auditorium, cafeteria, dance studio)	☐	☐	☐	☐	☐
c.	Fitness room for aerobic and/or strength training	☐	☐	☐	☐	☐
d.	Running track	☐	☐	☐	☐	☐
e.	Outdoor sports field (e.g., football or soccer)	☐	☐	☐	☐	☐
f.	Outdoor paved area (e.g., tennis courts, basketball courts, any paved area that can be used for active games like skipping or hopscotch)	☐	☐	☐	☐	☐
g.	Skating rink/arena	☐	☐	☐	☐	☐
h.	Indoor swimming pool	☐	☐	☐	☐	☐
i.	Secure change room lockers available for use during physical activity	☐	☐	☐	☐	☐
j.	Change rooms available for use before and after physical activity	☐	☐	☐	☐	☐
k.	Showers available for use before or after physical activity	☐	☐	☐	☐	☐
l.	Bicycle racks	☐	☐	☐	☐	☐
m.	**<u>If yes</u>**, are the racks in a secure area to avoid theft?	☐	☐	☐	☐	☐
n.	Grassy playground area	☐	☐	☐	☐	☐
o.	Playground equipment (e.g., climbing structures, swings)	☐	☐	☐	☐	☐
p.	Art room	☐	☐	☐	☐	☐
q.	Music room	☐	☐	☐	☐	☐

19. Do students have access to the following facilities where they can buy foods or drinks?

		Yes	*No*
a.	Cafeteria	☐	☐
b.	School shop	☐	☐
c.	Shops/fast food restaurants close to school	☐	☐
d.	Candy and potato chips vending machine	☐	☐
e.	Drinks vending machine (e.g., coke, soft drinks, orange juice)	☐	☐
f.	Milk vending machine/ milk program (e.g., milk, chocolate milk)	☐	☐

20. *Outside of school hours*,* does your school permit regular student access to the following?
**Outside of school hours means before and/or after school, evenings and weekends. Student access may occur via school-led, community-led or informal use.*

	Yes	*No*	*Don't know*	*N/A*
a. Gymnasium	☐	☐	☐	☐
b. Indoor facilities	☐	☐	☐	☐
c. Outdoor facilities (e.g., playing fields, paved activity areas, baseball diamond)	☐	☐	☐	☐
d. Equipment (e.g., basketballs)	☐	☐	☐	☐

21. *Outside of school hours, does your school allow community groups to use the school facilities?**
**Outside of school hours means before and/or after school, evenings and weekends.*

Yes ☐ No ☐ Don't know ☐

E. HEALTHY EATING

22. Does your school provide any of the following to promote the sale of healthy food? (Check all that apply)

	Cafeteria	*Snack bar/ School shop*	*Vending machine(s)*
a. Healthy food choices at a reasonable/subsidized price	☐	☐	☐
b. Healthy eating promotional materials (e.g., posters)	☐	☐	☐
c. Daily healthy eating specials	☐	☐	☐
d. Healthy eating cafeteria program (e.g., Eat Smart or independent program)	☐	☐	☐

23. Does your school ensure that all students, regardless of ability to pay, have access to fruits and vegetables?

- ☐ Yes, entire school year
- ☐ Yes, occasional/short term
- ☐ No

24. Does your school offer any of the following? (Check all that apply)

- ☐ Cooking classes
- ☐ Gardening (e.g., growing produce)
- ☐ Field trips to farms/farmers' markets
- ☐ Media literacy on special topics related to healthy eating (e.g., body image, eating disorders)
- ☐ Field trips to the local grocery store

25. During the past 12 months, did your school initiate/continue any of the following activities/programs at your school?

	Yes	*No*	*N/A*
a. Offered healthy food choices during breakfast program	☐	☐	☐
b. Offered healthy food choices during lunch program	☐	☐	☐
c. Offered healthy food choices in the cafeteria(s)	☐	☐	☐
d. Offered healthy food choices in the snack bar/school shop(s)	☐	☐	☐
e. Offered healthy food choices in the vending machine(s)	☐	☐	☐
f. Organized Nutrition Month activities	☐	☐	☐
g. Stopped the sale of junk food	☐	☐	☐
h. Held junk food free days	☐	☐	☐
i. Stopped the sale of sugar-sweetened beverages	☐	☐	☐

26. During the past 12 months, have any of the following items been sold as part of fundraising for any school organization?

	Yes	*No*	*N/A*
a. Chocolate candy	☐	☐	☐
b. Other candy	☐	☐	☐
c. Other junk food (e.g., popcorn)	☐	☐	☐
d. Soda pop or fruit drinks that are not 100% juice	☐	☐	☐
e. Sports drinks	☐	☐	☐
f. Cookies, crackers, cakes, pastries, or other baked goods that are not low in fat	☐	☐	☐
g. Fruits or vegetables	☐	☐	☐
h. 100% fruit juice or vegetable juice	☐	☐	☐
i. Low-fat cookies, crackers, cakes, pastries, or other low-fat baked goods	☐	☐	☐

F. NEIGHBORHOOD/COMMUNITY

27. How much of a problem are the following in the neighborhood where this school is located?

	Major problem	*Moderate problem*	*Minor problem*	*Not a problem*	*I don't know*
a. Tensions based on racial, ethnic, or religious differences	☐	☐	☐	☐	☐
b. Garbage, litter, or broken glass in the street or road, on the sidewalks, or in yards	☐	☐	☐	☐	☐
c. Selling or using drugs or excessive drinking in public	☐	☐	☐	☐	☐
d. Gangs	☐	☐	☐	☐	☐
e. Heavy traffic	☐	☐	☐	☐	☐
f. Vacant or shabby houses and buildings	☐	☐	☐	☐	☐
g. Crime in the neighborhood	☐	☐	☐	☐	☐
